# Data on the application of early coagulation support protocol in the management of major trauma patients

**DOI:** 10.1016/j.dib.2019.104768

**Published:** 2019-11-07

**Authors:** Maria Grazia Bocci, Giuseppe Nardi, Giovanni Veronesi, Maria Beatrice Rondinelli, Antonella Palma, Valentina Fiore, Erica De Candia, Maria Bianchi, Maddalena Maresca, Roberta Barelli, Alessandra Tersali, Antonio Maria Dell’Anna, Gennaro De Pascale, Salvatore Lucio Cutuli, Giovanna Mercurio, Anselmo Caricato, Domenico Luca Grieco, Massimo Antonelli, Emiliano Cingolani

**Affiliations:** a Dipartimento di Scienze dell'Emergenza, Anestesiologiche e della Rianimazione, Fondazione Policlinico Universitario A. Gemelli IRCCS, Rome, Italy; b Istituto di Anestesiologia e Rianimazione, Università Cattolica del sacro Cuore, Rome, Italy; c UOC Anestesia e Rianimazione, Ospedale Infermi, Rimini, Italy; d Centro Ricerche in Epidemiologia e Medicina Preventiva, Dipartimento di Medicina Clinica e Sperimentale, Università degli Studi dell’Insubria, Varese, Italy; e UOC Medicina Trasfusionale e Cellule Staminali, Azienda Ospedaliera San Camillo Forlanini, Rome, Italy; f UOC Anestesia e Rianimazione, CTO Azienda Ospedaliera dei Colli, Napoli, Italy; g UOC Anestesia e Rianimazione, Azienda Ospedaliera Vito Fazi, Lecce, Italy; h UOS Malattie Emorragiche e Trombotiche, Fondazione Policlinico Universitario A. Gemelli IRCCS, Rome, Italy; i Istituto di Medicina Interna e Geriatria, Università Cattolica del sacro Cuore, Rome, Italy; j UOC Emotrasfusione, Fondazione Policlinico Universitario A. Gemelli IRCCS, Rome, Italy; k Istituto di Ematologia, Università Cattolica del sacro Cuore, Rome, Italy; l UOC Anestesia e Rianimazione, Ospedale San Giovanni Calibita Fatebenefratelli, Rome, Italy; m UOSD Shock e Trauma, Azienda Ospedaliera San Camillo Forlanini, Rome, Italy

**Keywords:** Blood coagulation, Critical care, Fibrinogen, Haemorrhage, Italy, Propensity score, Sensitivity analysis, Trauma centres, AIS, anatomical injury score, ECS, early coagulation support, FFP, fresh frozen plasma, LOS-ICU, length of intensive care unit stay, LOS-hospital, length of hospital stay, MTP, massive transfusion protocol, PLT, platelets, pRBC, packed red blood cells

## Abstract

This article provides additional data on the application of early coagulation support protocol in the management of major trauma patients. Data come from a retrospective analysis reported in the article “Early coagulation support protocol: a valid approach in real-life management of major trauma patients. Results from two Italian centres” [1]. Data contain information about the relationship between differences in resource use and mortality outcomes, and patient demographic and clinical features at presentation. Furthermore, a comparison between resource consumption, the probability of multiple transfusions and the mortality outcomes among propensity-score matched patients is reported.

**Table:** Specifications Table

Subject	Critical Care and Intensive Care Medicine
Specific subject area	Haemostasis/coagulopathy
Type of data	Tables Graph
How data were acquired	Data were retrospectively acquired from the registry data of the emergency department of two Italian trauma centres.
Data format	Raw and analysed
Parameters for data collection	Overall cohort: patients who were consecutively admitted with major trauma who had, or were at risk of, active bleeding, managed according to the massive transfusion (2011–2012) or the early coagulation protocol (2013–2014). Study cohort: patients who, according to international guidelines, was considered at risk of requiring multiple transfusions.
Description of data collection	Data from all major trauma patients with ISS >15 who were admitted to the ICU were included into Trauma Centre's databases (overall cohort). Data from patients who met the inclusion criteria were included into the multicentre database (study cohort). These data were matched with blood bank registries and the amounts of packed red blood cells, fresh frozen plasma, and platelet units administered within 24-h after admission were recorded.
Data source location	Fondazione Policlinico Universitario Agostino Gemelli IRCCS (Rome, Italy) Azienda Ospedaliera San Camillo Forlanini (Rome, Italy)
Data accessibility	With the article (Supplementary material)
Related research article	Maria Grazia Bocci et al. Early coagulation support protocol: a valid approach in real-life management of major trauma patients. Results from two Italian centres. *Injury* (In Press)

**Table:** 

**Value of the Data** • These data, alongside with those reported in the related research article, show that the application of the ECS protocol guarantees early coagulation support in major trauma patients with high bleeding risk. • Basing on these data, ECS protocol may be adopted by clinicians in real-life management of major trauma patients. • These data can be used to design further prospective studies with the aim to standardize timing and dosing of fibrinogen in the application of ECS protocol in major trauma patients. • The present data give more strength to the results of the related research article confirming a reduction in the average blood product consumption associated with the application of ECS protocol.

## 1 Data

Among the 518 patients admitted into the participating centres due to major trauma (overall cohort), 235, who had, or were at risk of, active bleeding, matched one of the inclusion criteria (study cohort) and were enrolled in the study [1]. The stratified analysis is reported in Table 1 and Table 2, raw data are reported in Supplementary material (Table S1). Table 1 shows a significant reduction in the blood products consumption in all the age groups of patients treated with early coagulation support (ECS) protocol and a greater length of hospital stay (LOS-hospital) reduction in patients ≥ 40 years old compared to the younger ones (p-value = 0.001). In patients with traumatic brain injury (anatomical injury score – AIS head ≥4) the ECS group had less units of packed red blood cells (pRBC) and platelet (PLT) transfused and a shorter LOS-hospital, while no reduction in the number of fresh frozen plasma (FFP) units transfused or the length of intensive care unit stay (LOS-ICU) was recorded. A significant reduction in the LOS-ICU was also observed in older (>65 years) and in more severe patients (≥3 inclusion criteria) treated with ECS protocol. Table 2 shows no statistically differences in 28-day mortality between pre-ECS and ECS groups. The 24-h mortality rate was higher in patients with severe traumatic brain injury (AIS head ≥ 4; RR = 1.64) than in patients without traumatic brain injury (RR = 0.98).

**Table 1:** Mean difference in resources absorption (Δ) and relative 95% CI for critical patients (n = 235), between the ECS and the pre-ECS study periods, according to patients' characteristics at admission.

	Patients, n [pre-ECS:ECS]	pRBC, units 2h		FFP units, 24h		PLT units, 24h		LOS-ICU, days		LOS-hospital, days	
		Δ^a^ (IC 95%)	p-value^b^	Δ^a^ (IC 95%)	p-value^b^	Δ^a^ (IC 95%)	p-value^b^	Δ^a^ (IC 95%)	p-value^b^	Δa (IC 95%)	p-value^b^
**Age, years**											
18–39	45:37	−1.5 (−2.4; −0.5)	0.5	−1.8 (−2.6; −1)	0.7	−1.4 (−1.9; −0.8)	1.0	0.5 (−1; 1.9)	0.8	−6.4 (−9; −3.9)	0.001
40–64	44:52	−2.3 (−3.1; −1.4)		−1.8 (−2.5; −1)		−1.3 (−1.9; −0.6)		0.8 (−0.8; 2.4)		−13.5 (−16.1; −11)	
≥65	29:28	−2 (−2.9; −1)		−1.4 (−2.1; −0.8)		−1.4 (−1.9; −0.8)		−0.1 (−2.1; 1.9)		−10.3 (−13.6; −7.1)	
**AIS Head**											
<4	83:85	−1.5 (−2.1; −0.9)	0.1	−2.1 (−2.6; −1.6)	0.004	−1.4 (−1.8; −0.9)	0.5	0.8 (−0.3; 1.9)	0.9	−10.3 (−12.2; −8.4)	0.7
≥4	35:32	−2.6 (−3.7; −1.6)		−0.7 (−1.5; 0.2)		−1.1 (−1.8; −0.3)		0.9 (−1; 2.8)		−9.6 (−12.5; −6.8)	
**Inclusion criteria, n**											
1	67:67	−0.8 (−1.3; −0.2)	<.0001	−0.6 (−1; −0.2)	<.0001	−0.6 (−1; −0.3)	<.0001	1 (−0.2; 2.3)	<.0001	−11.1 (−13.1; −9.1)	0.1
2	30:23	−1.3 (−2.3; −0.3)		−2 (−2.7; −1.2)		−1.1 (−1.7; −0.5)		4.1 (1.9; 6.2)		−9 (−12.6; −5.5)	
≥3	21:27	−7.4 (−9.3; −5.5)		−6.2 (−7.8; −4.5)		−4.4 (−5.8; −3)		−4.4 (−6.8; −2)		−7.1 (−10.8; −3.5)	
**SBP** ≤ **90 mmHg**											
No	57:53	−0.7 (−1.3; 0)	<.0001	−0.8 (−1.3; −0.2)	<.0001	−0.9 (−1.3; −0.4)	0.02	−0.8 (−2.2; 0.7)	0.003	−15.3 (−17.6; −13)	<.0001
Yes	61:64	−3.1 (−3.9; −2.3)		−2.6 (−3.4; −1.9)		−1.7 (−2.3; −1.1)		2.2 (0.9; 3.5)		−5.4 (−7.5; −3.2)	
**BE < -6 mmol/L**											
No	51:41	−1.3 (−2; −0.5)	0.03	−1.5 (−2; −0.9)	0.2	−0.8 (−1.3; −0.3)	0.02	1.9 (0.5; 3.4)	0.02	−9.7 (−12.2; −7.3)	0.6
Yes	67:76	−2.4 (−3.2; −1.7)		−2.1 (−2.7; −1.4)		−1.7 (−2.2; −1.2)		−0.4 (−1.7; 0.9)		−10.7 (−12.7; −8.6)	
**Lactate** ≥ **5 mmol/L**											
No	80:83	−1 (−1.5; −0.5)	0.0004	−1 (−1.4; −0.6)	0.002	−0.6 (−0.9; −0.3)	0.0003	0.7 (−0.4; 1.8)	0.6	−11.6 (−13.5; −9.7)	0.002
Yes	38:34	−3.4 (−4.7; −2.2)		−2.9 (−4; −1.8)		−2.5 (−3.4; −1.5)		1.3 (−0.6; 3.2)		−6.1 (−9; −3.1)	
**Haemoglobin** ≤ **9 mg/dL**											
No	86:89	−1.8 (−2.4; −1.3)	0.5	−1.7 (−2.1; −1.2)	0.6	−1.4 (−1.7; −1)	0.2	2.3 (1.2; 3.4)	<.0001	−7.8 (−9.7; −6)	<.0001
Yes	32:28	−1.4 (−2.8; 0)		−1.3 (−2.5; −0.2)		−0.7 (−1.7; 0.2)		−3.3 (−5.4; −1.3)		−15.8 (−19; −12.6)	

AIS = anatomical injury score; BE = base excess; FFP = fresh frozen plasma; LOS-hospital = length of hospital stay; LOS-ICU = length of intensive care unit stay; PLT = platelets; pRBC = packed red blood cells; SBP = systolic blood pressure.

a Mean difference between the ECS and the pre-ECS study period with 95% CI from Poisson regression model. A negative number indicates a reduction in the resources absorption during the ECS period.

b p-value to test interaction between patients' characteristics and mean difference in resources absorption (F-test).

**Table 2:** Relative Risk (95% CI) of in-hospital mortality for critical patients admitted during the ECS period with respect to the pre-ECS period, according to patients' characteristics at admission.

	Patients, n [pre-ECS:ECS]	Day 0			Day 1–28			Day 0–28		
		Patients, n [pre-ECS:ECS]	RR^a^ (IC 95%)	p-value^b^	Patients, n [pre-ECS:ECS]	RR^a^ (IC 95%)	p-value^b^	Patients, n [pre-ECS:ECS]	RR^a^ (IC 95%)	p-value^b^
**Age, years**										
18–64	89:89	5:8	1.6 (0.52; 4.89)	0.7	9:8	0.92 (0.36; 2.39)	0.6	14:16	1.14 (0.56; 2.34)	0.9
≥65	29:28	3:3	1.04 (0.21; 5.13)		6:8	1.39 (0.48; 4)		9:11	1.27 (0.52; 3.05)	
**AIS head**										
<4	83:85	2:2	0.98 (0.14; 6.93)	0.6	6:8	1.3 (0.45; 3.75)	0.8	8:10	1.22 (0.48; 3.09)	1.0
≥4	35:32	6:9	1.64 (0.58; 4.61)		9:8	1.12 (0.43; 2.9)		15:17	1.24 (0.62; 2.48)	
**Inclusion criteria, n**										
1	67:60	0:5	NE	–	8:7	0.95 (0.34; 2.61)	0.7	8:12	1.5 (0.61; 3.67)	0.5
≥2	51:50	8:6	0.77 (0.27; 2.2)		7:9	1.26 (0.47; 3.37)		15:15	1.02 (0.5; 2.09)	

AIS = anatomical injury score; BE = base excess; NE = not estimable (no events); RR = relative risk; SBP = systolic blood pressure.

a Relative Risk of mortality for patients in the ECS study period vs. patients in the pre-ECS period (reference) with 95% CI, from Poisson regression model. A number lower than 1 indicates a reduction in the mortality risk for patients in the ECS with respect to the pre-ECS period.

b p-value to test interaction between patients' characteristics and mortality RR (F-test).

**Fig. 1:**
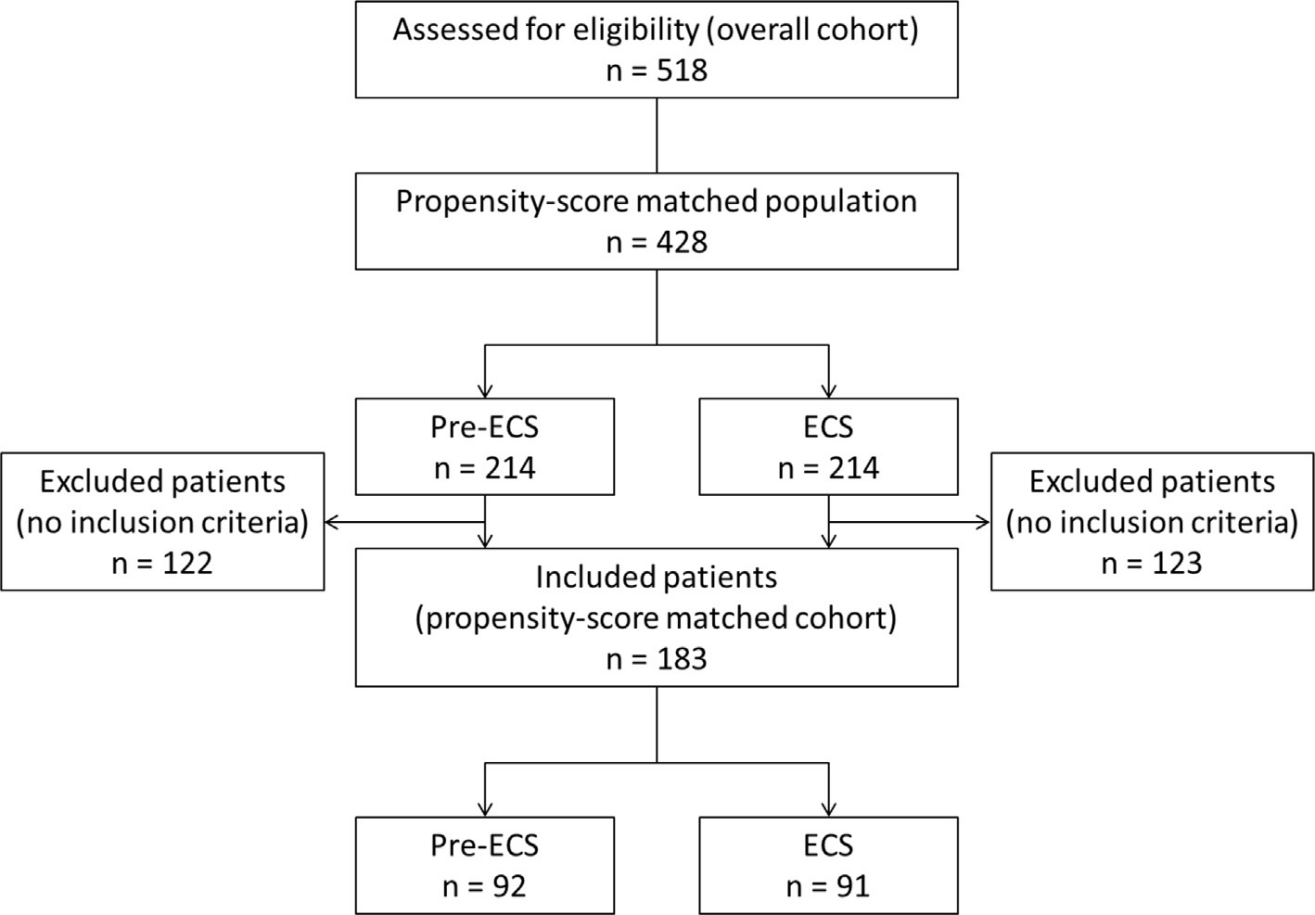
Patients disposition – Propensity-score matched cohort. ECS = early coagulation protocol.

Patients disposition and baseline characteristics of propensity-score matched patients are reported in Fig. 1 and Table 3. The propensity-score matched analysis is reported in Table 4 and shows a significantly lower use of pRBC, FFP, PLTs in patients treated with ECS protocol. Furthermore, in the ECS group were recorded a significant increase in LOS-ICU, and a decrease in LOS-hospital and mortality at day-zero. Raw data are reported in Supplementary material (Table S2).

**Table 3:** Demographic characteristics, clinical data, injury scores and laboratory parameters at hospital admission, by study period, among propensity-score matched patients.

	Propensity-score matched cohort (n = 183)		
	Pre ECS (n = 92)	ECS (n = 91)	p-value^a^
Inclusion criteria, n (25th, 75th percentiles)	1 (1, 2)	1 (1, 2)	0.9
Mean age, years (SD)	49.4 (21)	48.3 (19.6)	0.7
Men, n (%)	65 (70.7%)	71 (78%)	0.3
Mean SBP, mmHg (SD)	97.6 (31.5)	95.6 (33)	0.7
Median GCS (25th, 75th percentiles)	13 (7, 15)	14 (8, 15)	0.3
**Median ISS (25th, 75th percentiles)**	**34 (24.5, 41)**	**32 (19, 42)**	**0.4**
AIS head ≥ 4, n (%)	26 (28.3%)	25 (27.5%)	0.9
AIS chest ≥ 4, n (%)	39 (42.4%)	36 (39.6%)	0.7
**AIS abdomen** ≥ **4, n (%)**	**32 (34.8%)**	**21 (23.1%)**	**0.1**
**AIS pelvis and limbs** ≥ **4, n (%)**	**18 (19.6%)**	**31 (34.1%)**	**0.03**
AIS face ≥ 4, n (%)	13 (14.1%)	7 (7.7%)	0.2
AIS extremities ≥ 4, n (%)	4 (4.3%)	6 (6.6%)	0.5
Mean pH (SD)	7.3 (0.1)	7.3 (0.1)	0.2
Mean lactate, mmol/L (SD)	4.1 (2.3)	3.8 (2.8)	0.5
Mean BE, mmol/L (SD)	−6.8 (3.6)	−7 (3.6)	0.7
Mean fibrinogen, mg/dL (SD)	207.5 (89.9)	203.7 (108.9)	0.8
Mean INR (SD)	1.2 (0.3)	1.3 (0.4)	0.6
Mean haemoglobin, g/d (SD)	11.4 (2.6)	11.1 (2.6)	0.4
Mean PLT, x1000/dL (SD)	211 (83.4)	192.9 (75.4)	0.1

AIS = anatomical injury score; BE = base excess; GCS = Glasgow coma score; INR = international normalized ratio; ISS = injury severity score; PLT = platelets; SBP = systolic blood pressure.

a p-value for testing the null hypothesis of no difference in the patients' characteristics between the two study periods. Test statistic: chi-square, t-test and Wilcoxon rank test for dichotomic, continuous and discrete variables, respectively.

**Table 4:** Mean difference in resources absorption and relative risk of in-hospital mortality for propensity-score matched cohort between the ECS and the pre-ECS study periods.

	Study period		Mean difference^a^ (95% CI)	RR^b^ (95% CI)
	Pre ECS (n = 92)	ECS (n = 91)		
**Resources absorption, median (25**th**, 75**th **percentiles)**				
pRBC units 24 h	3.5 (0, 5.5)	1 (0, 5)	−1.01 (−1.57; −0.45)	–
FFP units 24 h	0 (0, 5)	0 (0, 4)	−0.69 (−1.15; −0.24)	–
PLT units 24 h	0 (0, 4)	0 (0, 0)	−0.89 (−1.26; −0.52)	–
LOS-ICU, days	9 (3, 18.5)	9 (3, 19)	2.57 (1.48; 3.66)	–
LOS-hospital, days	31 (14, 52)	30 (14, 43)	−7.72 (−9.49; −5.95)	–
**In-hospital mortality, n (%)**				
Day 0	6 (6.5%)	7 (7.7%)	–	1.18 (0.4; 3.51)
Day 1–28	14 (16.3%)	13 (15.5%)	–	0.95 (0.45; 2.02)
Day 0–28	20 (21.7%)	20 (22%)	–	1.01 (0.54; 1.88)

FFP = fresh frozen plasma; LOS-hospital = length of hospital stay; LOS-ICU = length of intensive care unit stay; PLT = platelets; pRBC = packed red blood cells; RR = relative risk.

a Mean difference between the ECS and the pre-ECS study period with 95% CI from Poisson regression model. A negative number indicates a reduction in the resources absorption during the ECS period.

b RR of mortality for patients in the ECS study period vs. patients in the pre-ECS period (reference) with 95% CI, from Poisson regression model. A number lower than 1 indicates a reduction in the mortality risk for patients in the ECS with respect to the pre-ECS period.

## 2 Experimental design, materials, and methods

Data of adult major trauma patients with, or at risk of, active bleeding, who were managed according to the massive transfusion protocol – MTP (years 2011–2012) or the ECS protocol (2013–2014) and were considered at risk of multiple transfusions, were retrospective collected with the aim to determine blood product consumption, length of stay, and in-hospital mortality.

A stratified analysis was performed in order to investigate whether differences in resource use and mortality between ECS and pre-ECS were related to patient demographic and clinical features at presentation, including age (18–40, 40–64, ≥65 years), severity of traumatic brain injury (head AIS <4 vs. ≥ 4), and major trauma severity (according to inclusion criteria). Stratified analyses were performed including a study period patients' feature interaction in separated Poisson models and by formally testing the null hypothesis of equal efficacy of the ECS protocol among categories of patients through an F test.

We defined “multiple transfused patients” as those experiencing four or more pRBC units during the first 24-h. The cut-off of four pRBC units represented the sample 75th percentile. We estimated the mean difference in transfused units and length of stay between ECS and pre-ECS from unadjusted Poisson models, using the delta methods to estimate the 95% CI for the mean difference. Findings were reinforced by replacing the Poisson model with a negative binomial distribution. In addition, we investigated the association between clinical features and the probability of multiple transfusion by means of univariate and multivariate logistic regression models.

To further control for any residual difference in clinical features between pre-ECS and ECS, we compared resource consumption, the probability of multiple transfusions and the mortality outcomes among propensity-score matched patients. The propensity score included the following variables: age, sex, ISS, AIS head, abnormal systolic blood pressure, blood base excess, lactate, haemoglobin, and platelets at hospital admission. The propensity score matching was performed using a standard macro.

All statistical analyses were performed using SAS 9.4 (SAS Institute, Cary NC).
